# Contribution of pudendal nerve injury to stress urinary incontinence in a male rat model

**DOI:** 10.1038/s41598-024-57493-1

**Published:** 2024-03-28

**Authors:** Shaimaa Maher, Daniel Gerber, Brian Balog, Lan Wang, Mei Kuang, Brett Hanzlicek, Tejasvini Malakalapalli, Cassandra Van Etten, Roger Khouri, Margot S. Damaser

**Affiliations:** 1 Department of Biomedical Engineering, Lerner Research Institute, Cleveland Clinic, 9500 Euclid Avenue ND20, Cleveland, OH 44195 USA; 2 Glickman Urological and Kidney Institute, Cleveland Clinic, Cleveland, OH USA; 3 Advanced Platform Technology Center, Louis Stokes Cleveland VA Medical Center, Cleveland, OH USA

**Keywords:** Neurology, Urology

## Abstract

Urinary incontinence is a common complication following radical prostatectomy, as the surgery disturbs critical anatomical structures. This study explored how pudendal nerve (PN) injury affects urinary continence in male rats. In an acute study, leak point pressure (LPP) and external urethral sphincter electromyography (EMG) were performed on six male rats with an intact urethra, the urethra exposed (UE), the PN exposed (NE), and after PN transection (PNT). In a chronic study, LPP and EMG were tested in 67 rats 4 days, 3 weeks, or 6 weeks after sham PN injury, PN crush (PNC), or PNT. Urethras were assessed histologically. Acute PNT caused a significant decrease in LPP and EMG amplitude and firing rate compared to other groups. PNC resulted in a significant reduction in LPP and EMG firing rate 4 days, 3 weeks, and 6 weeks later. EMG amplitude was also significantly reduced 4 days and 6 weeks after PNC. Neuromuscular junctions were less organized and less innervated after PNC or PNT at all timepoints compared to sham injured animals. Collagen infiltration was significantly increased after PNC and PNT compared to sham at all timepoints. This rat model could facilitate preclinical testing of neuroregenerative therapies for post-prostatectomy incontinence.

## Introduction

Stress urinary incontinence (SUI), characterized by the involuntary leakage of urine during activities involving physical exertion, poses a significant challenge following radical prostatectomy (RP)^1^. This surgical procedure, while often essential for the treatment of prostate cancer, gives rise to SUI with a prevalence of 2.5–90% of men^2,3^. In a recent prospective non-randomized trial that compared open retropubic radical prostatectomy (rPR) with robotic-assisted rPR, the incidence of urinary incontinence 12 months post-operatively was 21.3% for robotic-assisted rPR and 20.2% for open rPR^4^. Post-prostatectomy SUI (PPSUI) is the source of significant psychosocial burden, negatively impacting social relationships, emotional health, and physical exercise^5^.

The intricate mechanism underlying male urinary continence is essential to understand PPSUI. Urinary continence in men is maintained by proper function of the detrusor smooth muscle, the proximal intrinsic sphincter at the bladder neck, the external urethral sphincter (EUS), and the urethral suspensory mechanism (pubourethral ligaments)^6–8^. During RP, both the bladder neck and pubourethral ligaments are transected, and the bladder is then surgically anastomosed to the urethra. Therefore, post-prostatectomy continence is maintained primarily by the EUS, which is innervated by the pudendal nerve (PN)^9^.

Despite the ubiquity of PPSUI, its etiology is multifaceted and is not fully understood. PPSUI is likely caused by a combination of nerve damage (PN, inferior hypogastric plexus, pelvic nerves, or sphincteric branches from the cavernous nerves), direct damage to the EUS, and a decrease in functional urethral length^8,10–16^. While female stress urinary incontinence due to human vaginal delivery has been extensively modeled in animal studies^17^, the development of analogous models for PPSUI in males has remained an area of limited progress.

Studies on the effects of PN injury in males following RP have revealed the impact on the EUS, as reflected by atrophy with associated functional implications of reduced EUS contractility observed through transurethral ultrasound in cadavers^7^. In addition, a number of studies have demonstrated potential therapeutic avenues for enhancing continence parameters via electrical stimulation of the pelvic floor^18–22^. Opting for male laboratory rats as models for our study is based on their close similarities to humans in anatomy and physiology. This model not only makes scientific exploration easier but also emphasizes the ethical duty to conduct research with a dedication to advancing medical knowledge and improving human health^23^. In light of these considerations, the purpose of the present study is to better understand the roles of the PN and EUS in maintaining male urinary continence. By investigating the effects of both short-term and long-term PN injuries on male urinary continence, we aim to contribute to the knowledge base surrounding PPSUI. Establishment of a male rat model of PN injury would be useful for investigating neuro-regenerative strategies to treat PPSUI.

## Results

### Acute study

**Figure 1:**
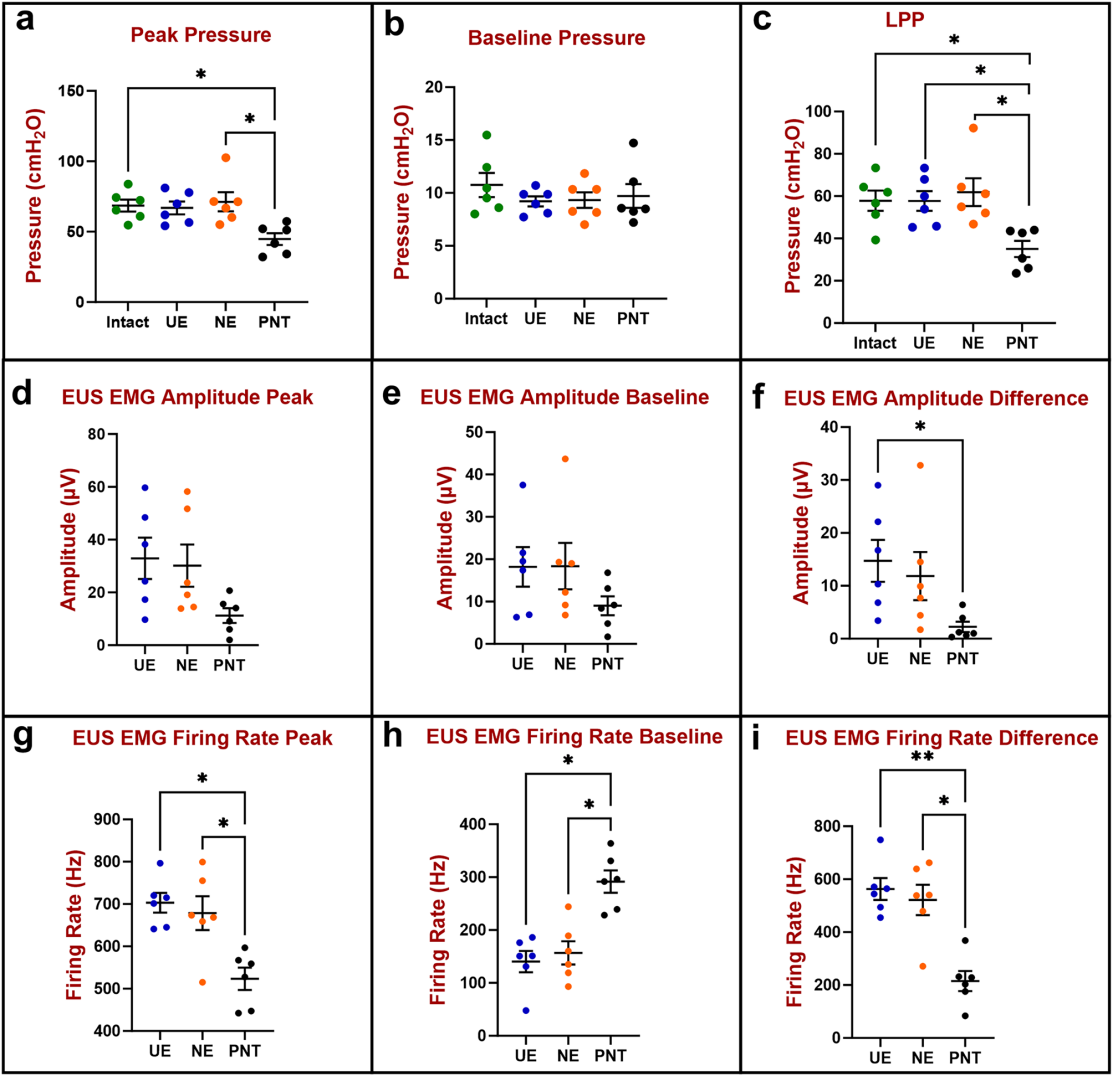
Urethral resistance to leakage in the acute study (n = 6) was evaluated by measuring peak and baseline bladder pressure during leak point pressure (LPP) testing with simultaneous external urethral sphincter electromyography (EUS EMG) recordings. The difference between peak (**a**) and baseline (**b**) bladder pressure is the LPP (**c**). EUS EMG was quantified as amplitude (**d**–**f**) and firing rate (**g**–**i**) at baseline (**e**,**h**) and peak (**d**,**g**) during LPP testing as well as the difference between them (**f**,**i**) which shows response to LPP testing. The scatter plots show individual data points, mean, and standard error of the mean (SEM). The Kruskal–Wallis test followed by Dunn’s multiple comparisons test was used to compare LPP (**a**–**c**), EUS EMG amplitude (**d**–**f**), and EUS EMG firing rate (**g**–**i**) between four groups: Intact (green), urethra exposed (UE, blue), nerve exposed (NE, orange) and pudendal nerve transected (PNT, black). *p < 0.05, **p < 0.01.

Six animals were subjected to a terminal study of repeated urethral neuromuscular functional recordings, including leak point pressure (LPP) and EUS electromyography (EMG) after different stages of dissection to expose the urethra and the PN and after PN transection (PNT). Peak bladder pressure was significantly lower in the PNT state (44.8 ± 4.2 cmH_2_O) compared to both the urethra intact (68.6 ± 4.2 cmH_2_O) and PN exposed (NE) states (71.2 ± 6.8 cmH_2_O; Fig. 1a). There were no significant differences between the urethra exposed (UE) state (66.9 ± 4.5 cmH_2_O) and the other stages of dissection. There was no significant change in baseline bladder pressure between intact (10.8 ± 1.1 cmH_2_O), UE (9.2 ± 0.5 cmH_2_O), NE (9.3 ± 0.7 cmH_2_O), and PNT (9.7 ± 1.1 cmH_2_O; Fig. 1b). Following PNT, LPP (35.0 ± 3.8 cmH_2_O), calculated as the difference between peak and baseline bladder pressure, was significantly decreased compared to NE (61.9 ± 6.6 cmH_2_O, p < 0.02), UE (57.7 ± 4.7 cmH_2_O, p < 0.05), and intact states (57.8 ± 4.8 cmH_2_O, p < 0.05), demonstrating decreased urethral resistance to leakage after PNT. In addition, there were no significant differences in LPP between intact, UE, and NE states (Fig. 1c).

There were no significant changes in baseline or peak EUS EMG amplitude during LPP testing between the UE (18.2 ± 4.7 μV, 32.9 ± 7.8 μV, respectively), NE (18.4 ± 5.5 μV, 30.18 ± 8.0 μV, respectively), and PNT (9.0 ± 2.2 μV, 11.3 ± 2.8 μV, respectively) states (Fig. 1d,e). After PNT, EUS EMG amplitude response to LPP testing (2.2 ± 1.0 μV), calculated as the difference between peak and baseline, was significantly decreased compared to the UE state (14.7 ± 4.0 μV, p < 0.02), demonstrating decreased EUS response to LPP testing (Fig. 1f).

Peak EUS EMG firing rate during LPP testing was significantly decreased in the PNT state (523 ± 26 Hz) compared to both the NE (679 ± 40 Hz) and UE states (703 ± 23 Hz; Fig. 1g). In contrast, baseline EUS EMG firing rate during LPP testing was significantly increased in the PNT state (292 ± 21 Hz) compared to both the NE (157 ± 22 Hz) and UE states (141 ± 20 Hz; Fig. 1h). After PNT, EUS EMG firing rate response to LPP testing (215 ± 38 Hz), calculated as the difference between peak and baseline, was also significantly decreased compared to the NE (522 ± 57 Hz, p < 0.03) and UE (563 ± 41 Hz, p < 0.008; Fig. 1i) states.

### Chronic study

Sixty-seven animals were randomly assigned to PN crush (PNC), PNT, or sham nerve injury (sham) groups. Due to surgical complications, 64 animals survived to their planned terminal study date and were able to provide meaningful data: 17 animals in the 4-day group (8 Sham, 9 PNC), 18 in the 3-week group (10 Sham, 8 PNC), and 29 in the 6-week group (10 Sham, 10 PNC, 9 PNT).

**Figure 2:**
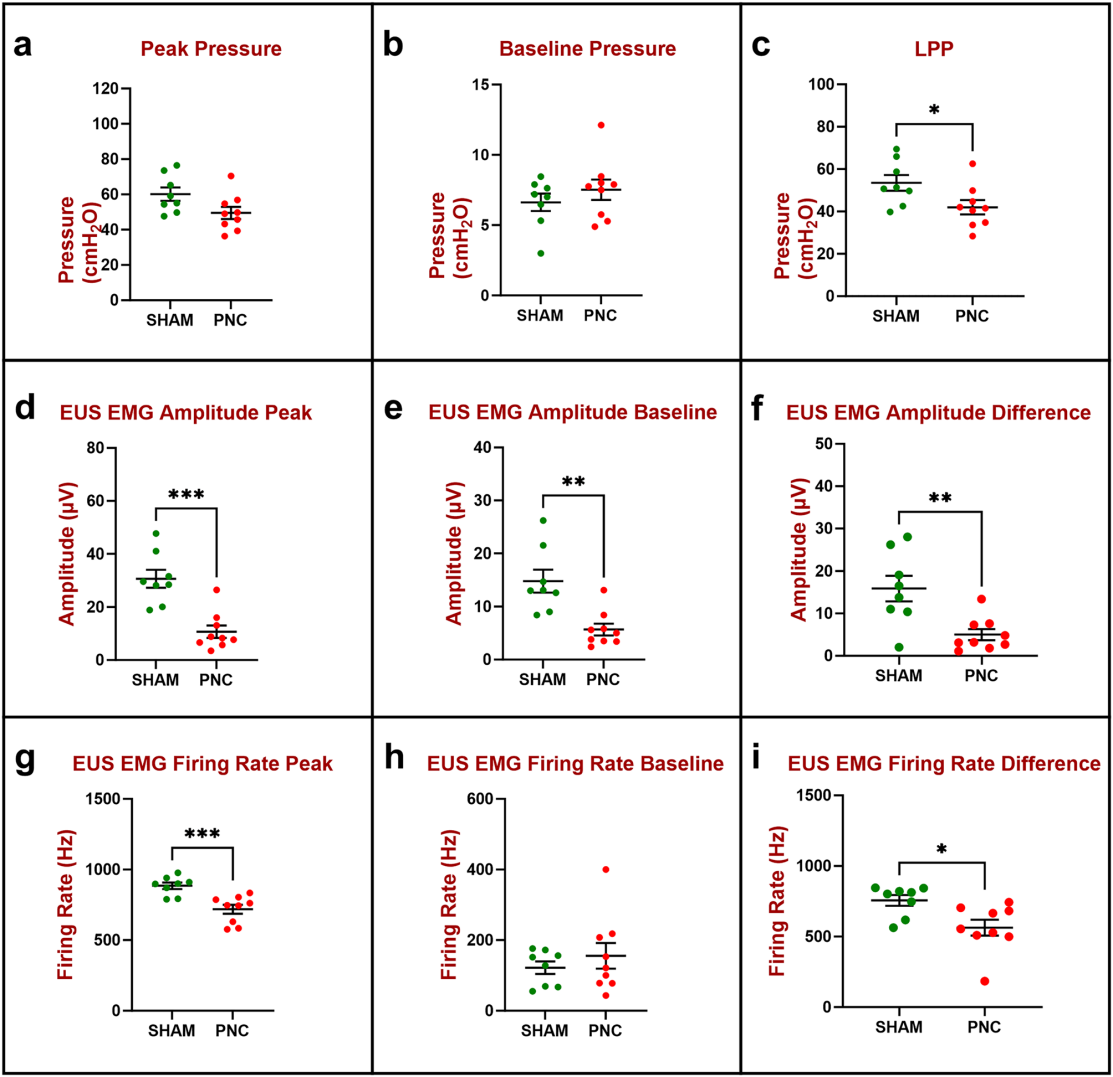
Urethral resistance to leakage in the chronic study 4 days after injury (n = 8 Sham, green and n = 9 PNC, red) was evaluated by measuring peak and baseline bladder pressure during leak point pressure (LPP) testing with simultaneous external urethral sphincter electromyography (EUS EMG) recordings. The difference between peak (**a**) and baseline (**b**) bladder pressure is the LPP (**c**). EUS EMG was quantified as amplitude (**d**–**f**) and firing rate (**g**–**i**) at baseline (**e**,**h**) and peak (**d**,**g**) during LPP testing as well as the difference between them (**f**,**i**) which shows response to LPP testing. The scatter plot graphs show individual data points, mean, and standard error of the mean (SEM). Unpaired two-tailed *t*-test was used to compare LPP (**a**–**c**), EUS EMG amplitude (**d**–**f**), and EUS EMG firing rate (**g**–**i**) between two groups (Sham, PNC). *p < 0.05, **p < 0.01, and ***p < 0.001. *PNC* pudendal nerve crush.

Four days after PNC (Fig. 2), peak (49.5 ± 3.4 cmH_2_O; Fig. 2a) and baseline bladder pressure (7.5 ± 0.7 cmH_2_O; Fig. 2b) were not significantly different from sham injured animals (60.2 ± 3.8 cmH_2_O, 6.6 ± 0.6 cmH_2_O, respectively). Peak (10.7 ± 2.3 μV; Fig. 2d) and baseline EUS EMG amplitude (5.7 ± 1.1 μV; Fig. 2e) during LPP testing were significantly decreased in the PNC group compared to sham injured animals (30.7 ± 3.4 μV, 14.8 ± 2.1 μV, respectively). Peak EUS EMG firing rate during LPP testing was significantly decreased in the PNC group (719 ± 32 Hz; Fig. 2g) compared to sham injured animals (885 ± 23 Hz); while baseline EUS EMG firing rate did not show a significant difference between PNC (156 ± 36 Hz; Fig. 2h) and sham injured animals (122 ± 18 Hz; Fig. 2). Although peak and baseline values were not always significantly different from sham 4 days after PNC, their difference expressed as bladder pressure (42.0 ± 3.3 cmH_2_O; Fig. 2c), EUS EMG amplitude (5.0 ± 1.3 μV; Fig. 2f), and EUS EMG firing rate (563 ± 56 Hz; Fig. 2i) response to LPP testing were all significantly decreased 4 days after PNC compared to sham injured animals (53.5 ± 3.7 cmH_2_O, 15.9 ± 3.0 μV, 756 ± 38 Hz, respectively), demonstrating a reduction in urethral response to LPP testing after PNC.

**Figure 3:**
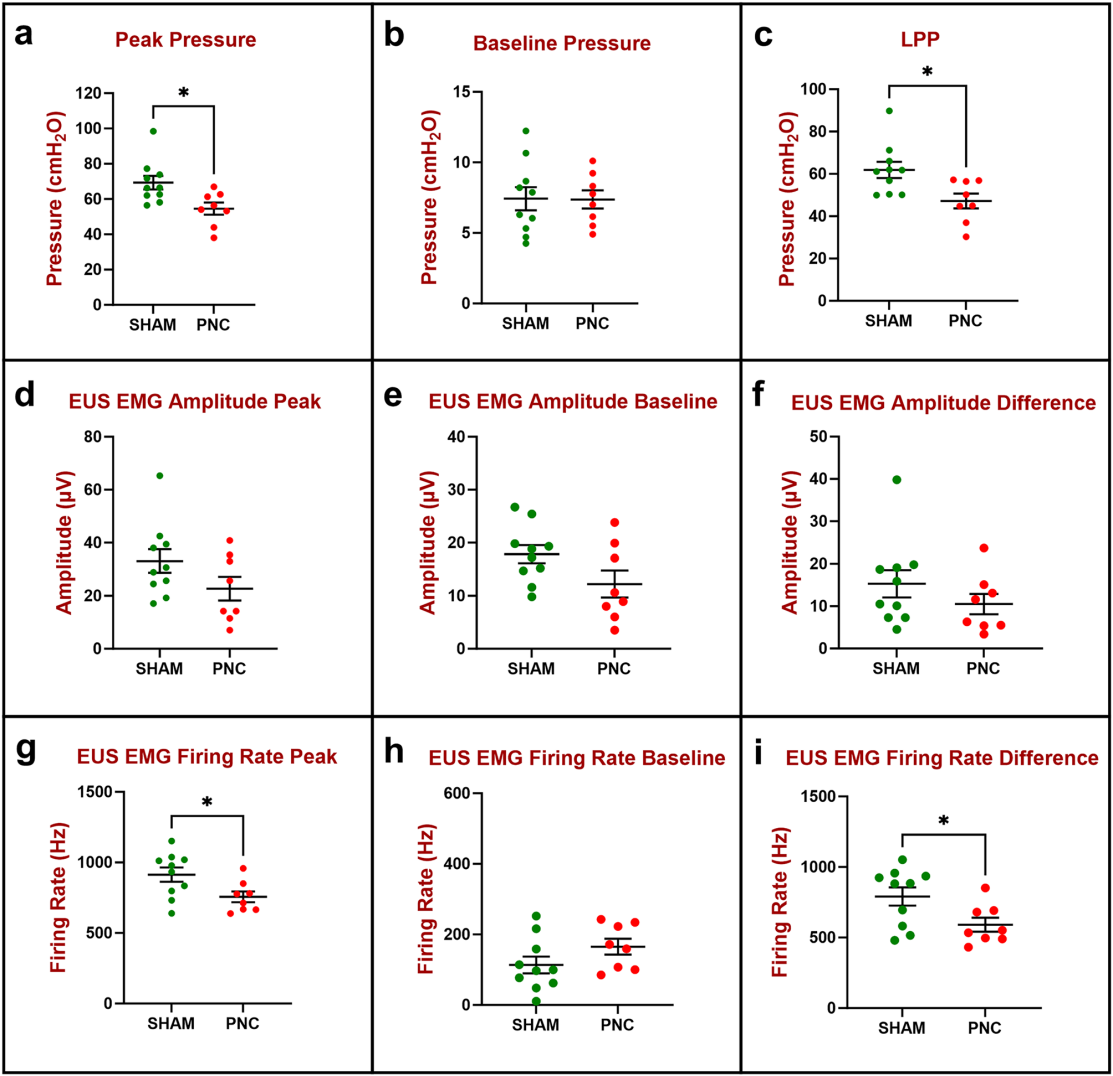
Urethral resistance to leakage in the chronic study 3 weeks after injury (n = 10 Sham, green and n = 8 PNC, red) was evaluated by measuring peak and baseline bladder pressure during leak point pressure (LPP) testing with simultaneous external urethral sphincter electromyography (EUS EMG) recordings. The difference between peak (**a**) and baseline (**b**) bladder pressure is the LPP (**c**). EUS EMG was quantified as amplitude (**d**–**f**) and firing rate (**g**–**i**) at baseline (**e**,**h**) and peak (**d**,**g**) during LPP testing as well as the difference between them (**f**,**i**) which shows response to LPP testing. The scatter plot graphs show individual data points, mean, and standard error of the mean (SEM). Unpaired two-tailed *t*-test was used to compare LPP (**a**–**c**), EUS EMG amplitude (**d**–**f**), and EUS EMG firing rate (**g**–**i**) between two groups (Sham, PNC). *p < 0.05. *PNC* pudendal nerve crush.

Three weeks after PNC (Fig. 3), peak bladder pressure (54.6 ± 3.4 cmH_2_O; Fig. 3a) during LPP testing was significantly decreased compared to sham injured animals (69.3 ± 3.9 cmH_2_O); while baseline bladder pressure did not show a significant difference between the PNC (7.4 ± 0.6 cmH_2_O; Fig. 3b) and sham injured animals (7.4 ± 0.8 cmH_2_O). Peak (22.7 ± 4.5 μV; Fig. 3d) and baseline EUS EMG amplitude (12.2 ± 2.5 μV; Fig. 3e) during LPP testing 3 weeks after PNC were not significantly different from sham injured animals (33.1 ± 4.5 μV, 17.9 ± 1.7 μV, respectively). Peak EUS EMG firing rate during LPP testing was significantly decreased 3 weeks after PNC (757 ± 38 Hz; Fig. 3g) compared to sham injured animals (914 ± 50 Hz); while baseline EUS EMG firing rate did not show a significant difference between the PNC (165 ± 22 Hz; Fig. 3h) and sham injured animals (114 ± 24 Hz; Fig. 3). Nonetheless, LPP (47.2 ± 3.5 cmH_2_O; Fig. 3c) and EUS EMG firing rate response to LPP testing (591 ± 49 Hz; Fig. 3i) were significantly decreased 3 weeks after PNC compared to sham injured animals (61.9 ± 3.8 cmH_2_O and 791 ± 65 Hz, respectively), demonstrating reduced urethral response at this timepoint. EUS EMG amplitude response to LPP testing was not significant different between PNC (10.5 ± 2.4 μV; Fig. 3f) and sham (15.3 ± 3.2.0 μV) groups 3 weeks after injury.

**Figure 4:**
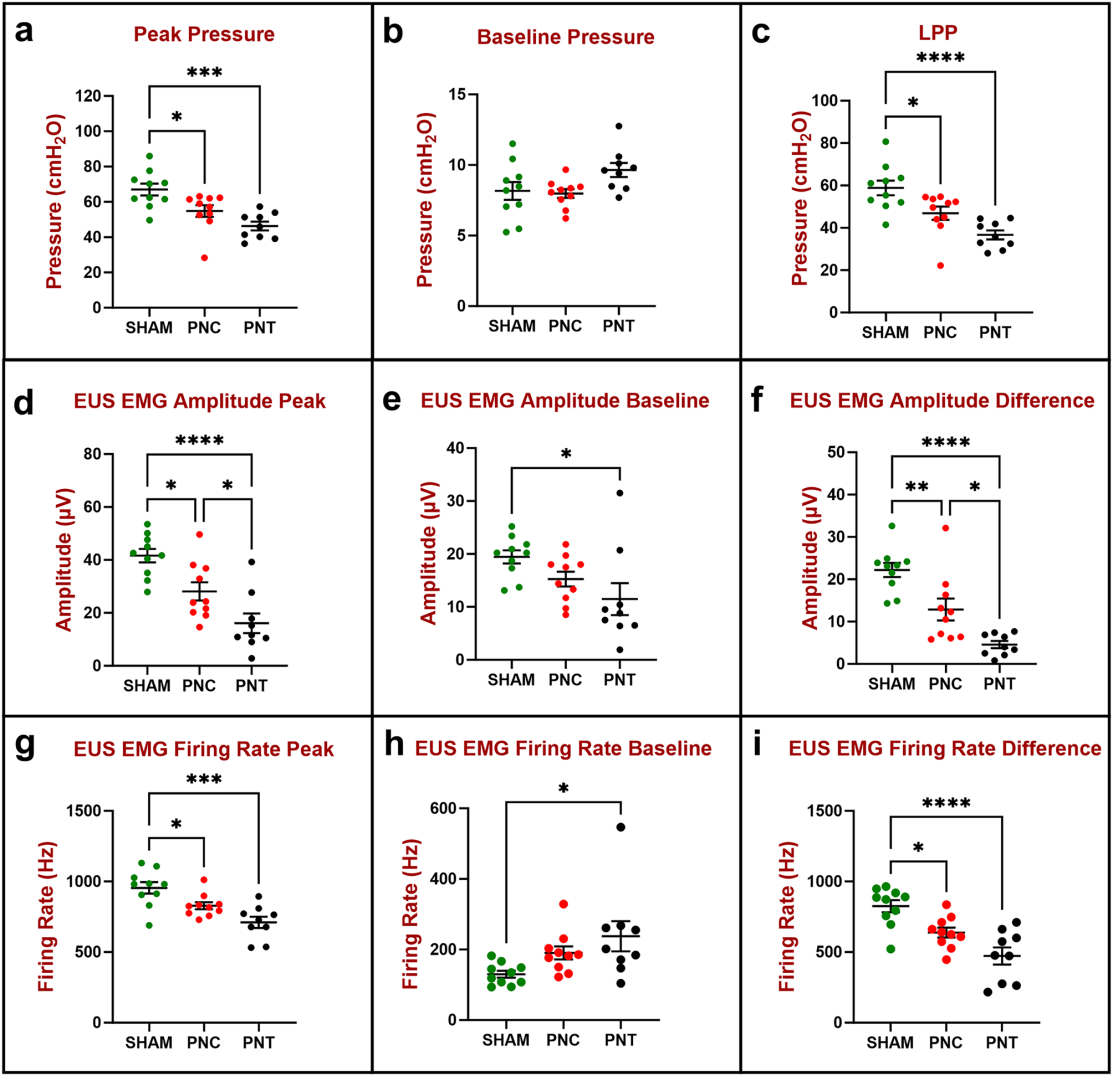
Urethral resistance to leakage in the chronic study 6 weeks after injury (n = 10 Sham, green; n = 10 PNC, red; and n = 9 PNT, black) was evaluated by measuring peak and baseline bladder pressure during leak point pressure (LPP) testing with simultaneous external urethral sphincter electromyography (EUS EMG) recordings. The difference between peak (**a**) and baseline (**b**) bladder pressure is the LPP (**c**). EUS EMG was quantified as amplitude (**d**–**f**) and firing rate (**g**–**i**) at baseline (**e**,**h**) and peak (**d**,**g**) during LPP testing as well as the difference between them (**f**,**i**) which shows response to LPP testing. The scatter plot graphs show individual data points, mean, and standard error of the mean (SEM). Ordinary one-way analysis of variance (ANOVA) followed by Tukey’s multiple comparisons test was used to compare LPP (**a**–**c**), EUS EMG amplitude (**d**–**f**), and EUS EMG firing rate (**g**–**i**) between three groups (Sham, PNC, PNT). *p < 0.05, **p < 0.01, ***p < 0.001, and ****p < 0.0001. *PNC* pudendal nerve crush, *PNT* pudendal nerve transected.

Peak bladder pressure 6 weeks after PNC (54.9 ± 3.3 cmH_2_O) and PNT (46.4 ± 2.5 cmH_2_O; Fig. 4a) during LPP testing were significantly decreased compared to sham injured animals (67.0 ± 3.3 cmH_2_O; Fig. 4). Baseline pressures did not show a significant difference between the PNC (8.0 ± 0.3 cmH_2_O), PNT (9.6 ± 0.5 cmH_2_O), and sham injured animals (8.2 ± 0.6 cmH_2_O; Fig. 4b) 6 weeks after injury. Peak EUS EMG amplitude during LPP testing was significantly decreased in the PNT group (16.1 ± 3.7 μV) compared to PNC (28.1 ± 3.4 μV) and sham injured animals (41.6 ± 2.5 μV; Fig. 4d). In addition, peak EUS EMG amplitude 6 weeks after PNC was significantly decreased during LPP testing compared to the sham group (Fig. 4d). Baseline EUS EMG amplitude during LPP testing 6 weeks PNT (11.5 ± 3.0 μV) was significantly decreased compared to sham injured animals (19.5 ± 1.2 μV; Fig. 4e). In contrast, there was no significant difference compared to the PNC group (15.3 ± 1.4 μV; Fig. 4e).

Peak EUS EMG firing rate during LPP testing was significantly decreased 6 weeks after PNT (710 ± 40 Hz) and PNC (828 ± 25 Hz) compared to sham injured animals (955 ± 41 Hz; Fig. 4g). Baseline EUS EMG firing rate was significantly increased 6 weeks after PNT (238 ± 43 Hz) compared to sham injured animals (130 ± 10 Hz). In contrast, there was no significant difference compared to the PNC group (190 ± 19 Hz). Six weeks after PNC, LPP (46.9 ± 3.1 cmH_2_O; Fig. 4c), EUS EMG amplitude (12.9 ± 2.6 μV; Fig. 4f), and EUS EMG firing rate (638 ± 35 Hz; Fig. 4i) response to LPP testing were all significantly decreased compared to sham injured animals (58.9 ± 3.5 cmH_2_O, 22.2 ± 1.7 μV, 825 ± 43 Hz, respectively). Likewise, 6 weeks after PNT, LPP (36.7 ± 2.1 cmH_2_O; Fig. 4c), EUS EMG amplitude (4.6 ± 0.9 μV; Fig. 4f), and EUS EMG firing rate (473 ± 61 Hz; Fig. 4i) response to LPP testing were all significantly decreased compared to sham, demonstrating consistent decreased neuromuscular response to LPP testing at all time points. In addition, EUS EMG amplitude 6 weeks after PNT was significantly decreased compared to PNC.

### Histological analysis

#### Masson’s trichrome stain

**Figure 5:**
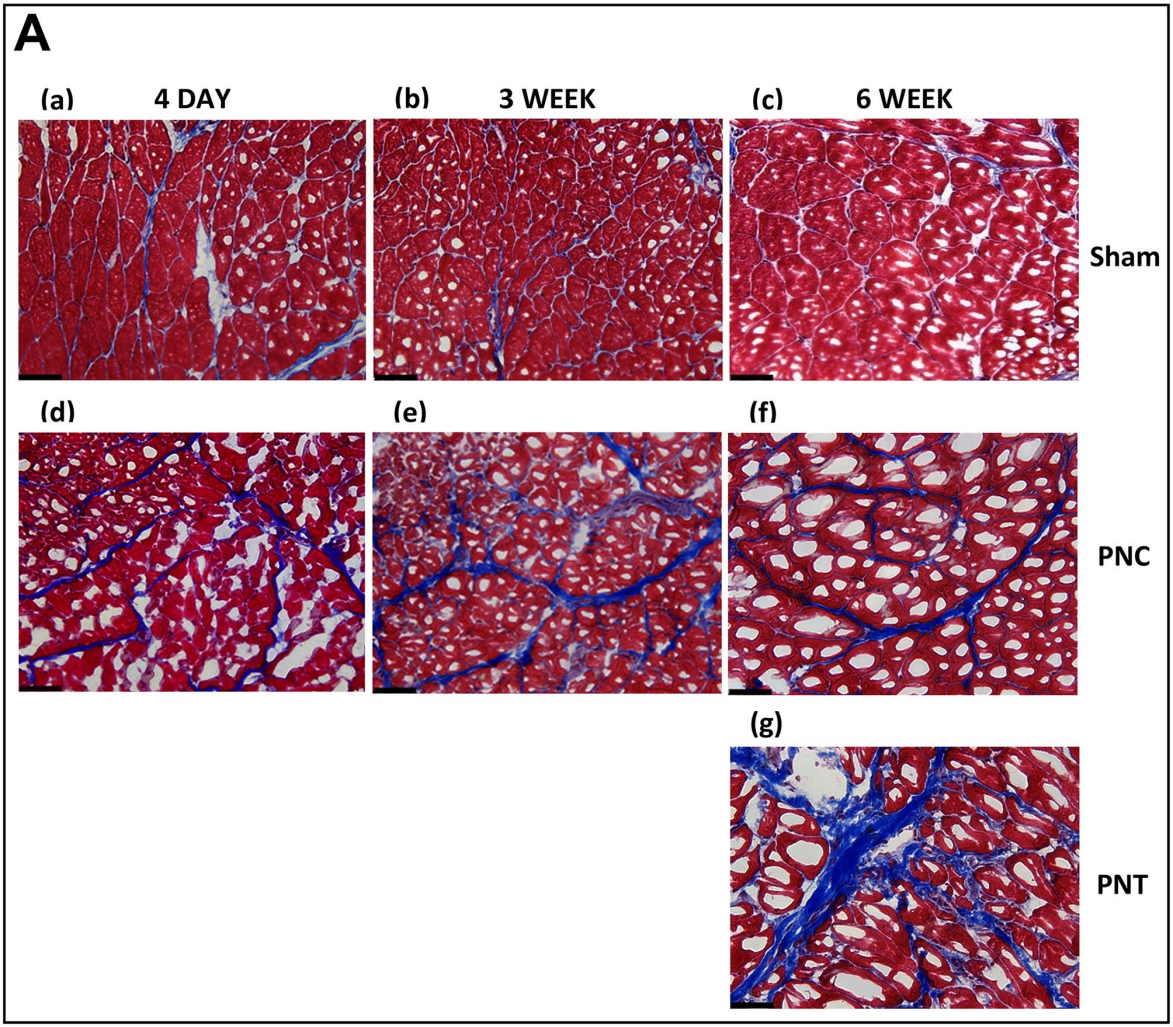

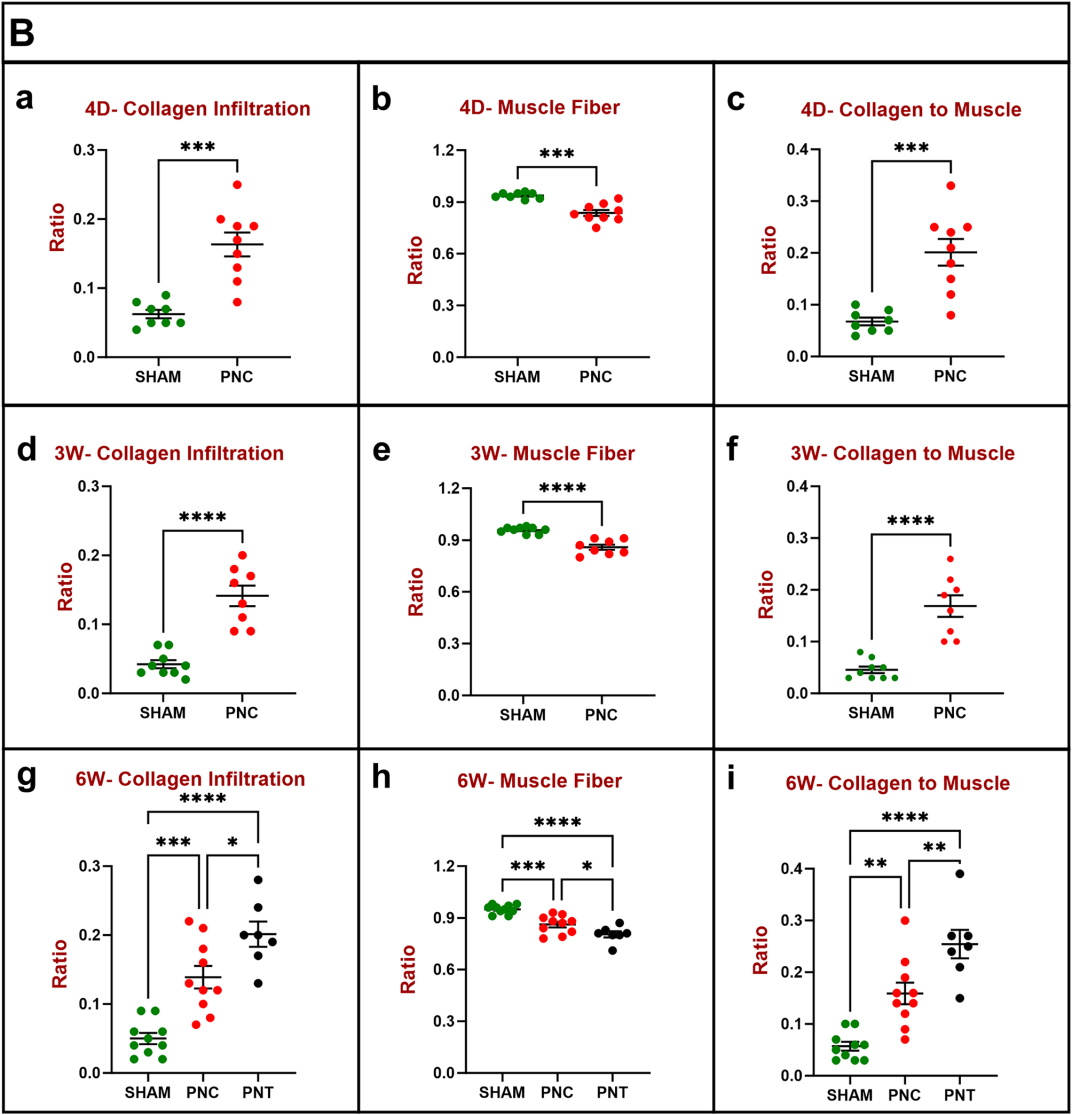
Urethral Masson’s trichrome stain results for chronic study. (**A**) Representative images of Masson’s trichrome-stained sections of urethral cross-sections. 4 days (1st column), 3 weeks (2nd column) and 6 weeks (3rd column). After sham injury (1st row), the images depict densely packed striated muscle fibers with minimal collagen infiltration. After pudendal nerve crush (PNC; 2nd row), there are atrophied striated muscle fibers with increased collagen infiltration. After pudendal nerve transection (PNT; 3rd row), striated muscle fibers of the EUS are atrophied and are accompanied by increased collagen infiltration. (**B**) Ratio of collagen (**a**,**d**,**g**) and muscle (**b**,**e**,**h**) to total area, and ratio of collagen to muscle (**c**,**f**,**i**) at each time point. Histology was assessed 4 days [(**a**–**c**) (n = 8 Sham, green and n = 9 PNC, red)], 3 weeks [(**d**–**f**) (n = 9 Sham, green and n = 8 PNC, red)], and 6 weeks [(**g**–**i**) (n = 10 Sham, green; n = 10 PNC, red; and n = 7 PNT, black)] after injury. The scatter plot graphs show individual data points, mean, and standard error of the mean (SEM). Unpaired two-tailed *t*-test was used to compare outcomes between the sham and PNC groups for both the 4-day and 3-week time points. Ordinary one-way analysis of variance (ANOVA) followed by Tukey’s multiple comparisons test was used to compare outcomes among the sham, PNC, and PNT groups at the 6-week time point. *p < 0.05, **p < 0.01. ***p < 0.001, ****p < 0.0001.

Qualitative analysis of the EUS in Masson’s trichrome stained urethral sections in the sham group at all timepoints after injury revealed typical morphology characterized by densely packed, striated muscle fibers separated by fine lines of connective tissue (Fig. 5Aa–c). Notably, there was minimal to no detectable collagen infiltration between the muscle fibers of the EUS in tissue sections from sham animals. Conversely, at all timepoints after PNC, the urethral striated muscle fibers were significantly atrophied, degenerated, accompanied by an expansion of the interstitial spaces between them, and infiltrated with collagen (Fig. 5Ad–f). In the PNT group 6 weeks after injury, the muscle tissue was most atrophied, with a lower muscle tissue area and a higher interstitial space, as well as higher collagen infiltration (Fig. 5Ag).

Quantitative analysis of the EUS in Masson’s trichrome stained urethral sections demonstrated a significant increase in collagen area relative to total cross-section area and a decrease in muscle area relative to total area in both the PNC and PNT groups 4 days, 3 weeks, and 6 weeks after injury compared to sham animals (Fig. 5B). Four days after injury, the collagen infiltration ratio was significantly increased for PNC (0.16 ± 0.02) compared to sham (0.06 ± 0.01, p = 0.0001; Fig. 5Ba). The muscle area ratio was significantly decreased for PNC (0.84 ± 0.02) compared to sham (0.94 ± 0.01, p = 0.0001; Fig. 5Bb). The collagen fiber-to-muscle tissue ratio was also significantly increased for PNC (0.20 ± 0.03) compared to sham (0.07 ± 0.01, p = 0.0003; Fig. 5Bc), indicating urethral fibrosis and atrophy of the EUS.

Three weeks after injury, the collagen infiltration ratio was significantly increased for PNC (0.14 ± 0.02) compared to sham (0.04 ± 0.01, p < 0.0001; Fig. 5Bd). The muscle area ratio was significantly decreased for PNC (0.86 ± 0.02) compared to sham (0.96 ± 0.01, p < 0.0001; Fig. 5Be). The collagen fiber-to-muscle tissue ratio was also significantly increased for PNC (0.17 ± 0.02) compared to sham (0.05 ± 0.01, p < 0.0001; Fig. 5Bf).

Six weeks after injury, the collagen infiltration ratio for sham (0.05 ± 0.01) was significantly lower than PNC (0.14 ± 0.02, p = 0.0003) and PNT (0.20 ± 0.02, p < 0.0001; Fig. 5Bg). Additionally, the PNT group had significantly higher collagen infiltration ratio compared to the PNC group (p = 0.0181). The muscle area ratio was significantly increased for sham (0.95 ± 0.01) compared to both PNC (0.86 ± 0.02, p = 0.0003) and PNT (0.80 ± 0.02, p < 0.0001; Fig. 5Bh). Moreover, the PNC group had higher muscle area ratio than the PNT group (p = 0.0333). The collagen fiber-to-muscle tissue ratio for sham (0.06 ± 0.01) was significantly lower than PNC (0.16 ± 0.02, p = 0.0015) and PNT (0.25 ± 0.03, p < 0.001; Fig. 5Bi), while the PNC group had significantly lower collagen fiber-to-muscle tissue ratio than the PNT group (p = 0.007).

#### Neuromuscular junction immunostaining

**Figure 6:**
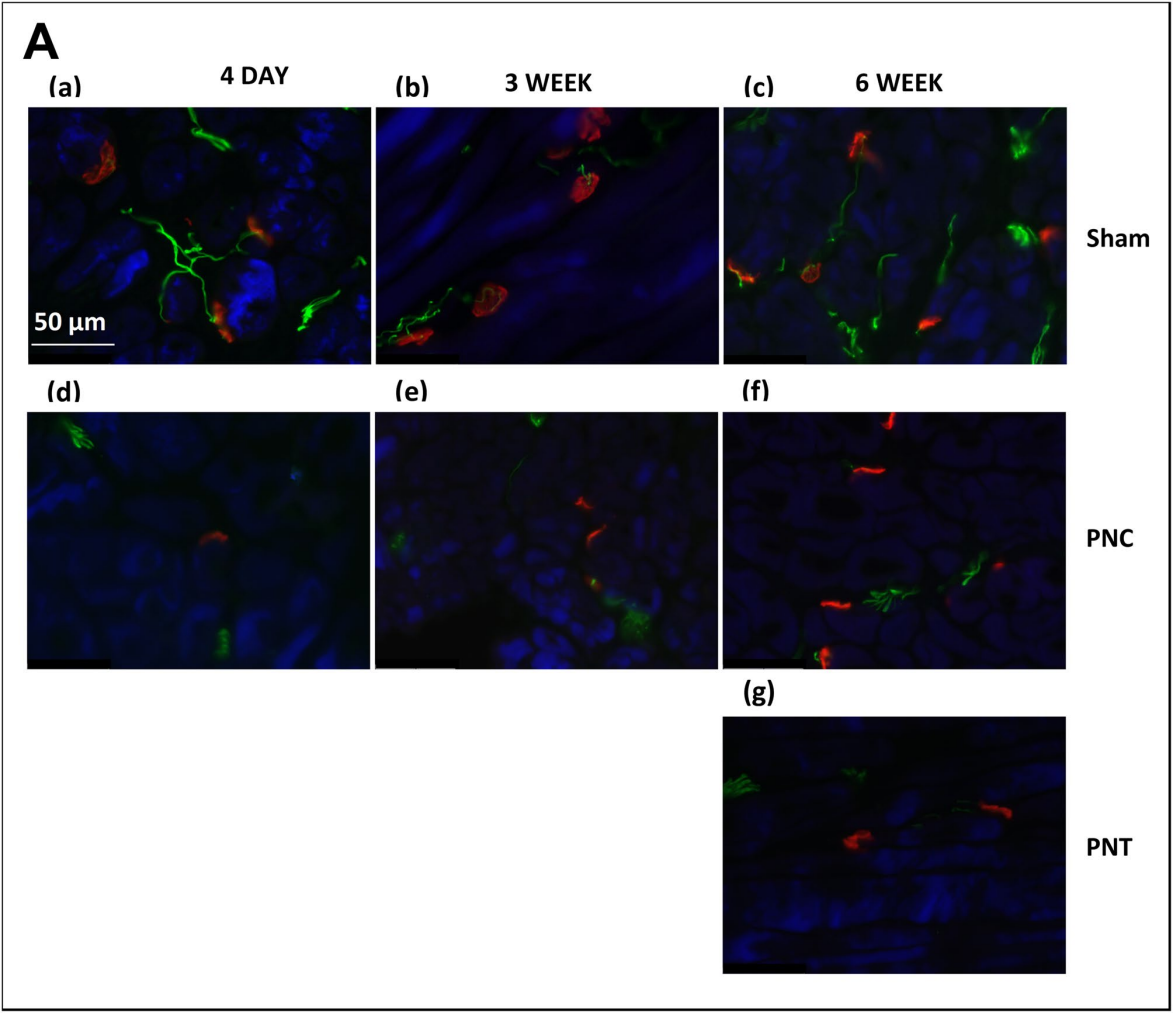

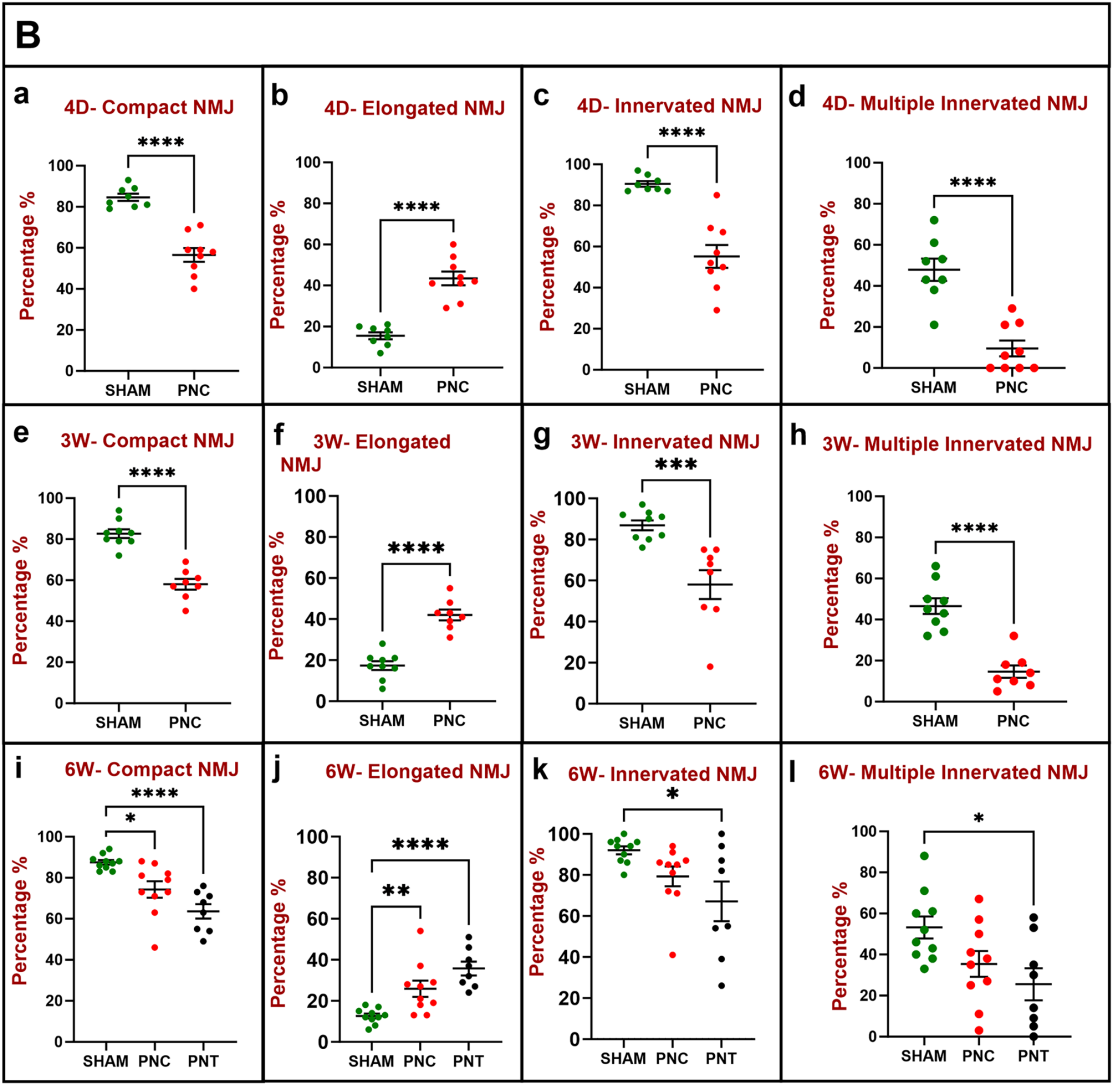
Urethral neuromuscular junction (NMJ) immunostaining results for chronic study. (**A**) Representative images of immunofluorescence-stained sections of urethral cross-sections. 4 days (1st column), 3 weeks (2nd column) and 6 weeks (3rd column). After sham injury (1st row), the images demonstrate well-defined, organized motor endplates [compact NMJ; (**a**–**c**)] accompanied by well innervated NMJ. After pudendal nerve crush (PNC; 2nd row), the NMJ became less organized and less well innervated [elongated NMJ; (**d**–**f**)]. After pudendal nerve transection (PNT; 3rd row), the images display less organization and less innervation (**g**). Green color indicates neurofilament, red color indicates NMJ, and blue color indicates striated muscle. (**B**) EUS NMJ quantitative assessments based on percentage of the specified criteria (compact, elongated, innervated, and multiple innervated) to total NMJ in 12 different fields per each specimen. Percentage compact NMJs (**a**,**e**,**i**), elongated NMJs (**b**,**f**,**j**), innervated NMJs (**c**,**g**,**k**), and multiple innervated NMJs (**d**,**h**,**l**) at each time point. Data presented at 4 days [(**a**–**d**) (n = 8 Sham, green and n = 9 PNC, red)], 3 weeks [(**e**–**h**) (n = 9 Sham, green and n = 8 PNC, red)], and 6 weeks [(**i**–**l**) (n = 10 Sham, green; n = 10 PNC, red; and n = 7 PNT, black)]. The scatter plot graphs show individual data points, mean, and standard error of the mean (SEM). Unpaired two-tailed *t*-test was used to compare outcomes between the sham and PNC groups 4 days and 3 weeks after injury. Ordinary one-way analysis of variance (ANOVA) followed by Tukey’s multiple comparisons test was used to compare outcomes among the sham, PNC, and PNT groups at the 6 weeks after injury. *p < 0.05, **p < 0.01, ***p < 0.001, ****p < 0.0001.

Qualitative assessments of neuromuscular junction (NMJ) in the EUS for sham groups 4 days, 3 weeks, and 6 weeks after injury demonstrated well-defined, organized motor endplates (compact NMJ; Fig. 6Aa–c) that were innervated by a single straight thick individual axon or multiple axons (Innervated NMJ) or were innervated by multiple axons (multiple innervated NMJ). Conversely, at all timepoints after PNC (Fig. 6Ad–f) and PNT (Fig. 6Ag), motor endplates were less organized (elongated), and fewer were innervated with axons. In the PNT group 6 weeks after injury, the innervating axons were thinner than sham and PNC groups (Fig. 6Ag).

Four days after PNC, the percentage of compact NMJs (56.6 ± 3.3%) was significantly decreased compared to the sham group (84.6 ± 1.8%, p < 0.0001; Fig. 6Ba), indicating NMJ denervation. The percentage of elongated NMJs was significantly increased in the PNC group (43.4 ± 3.3%) compared to sham injured animals (15.5 ± 1.7%, p < 0.0001; Fig. 6Bb), confirming denervation of NMJs in the EUS. Additionally, the innervation of NMJs was significantly decreased 4 days after PNC (55.2 ± 5.6%) compared to sham rats (90.5 ± 1.4%, p < 0.0001; Fig. 6Bc). Specifically, innervation of NMJ by multiple axons was decreased 4 days after PNC (9.6 ± 3.8%) compared to sham rats (47.9 ± 5.4%, p < 0.0001; Fig. 6Bd).

Three weeks after PNC, the percentage of compact NMJs (58.0 ± 2.6%) remained significantly decreased compared to sham rats (82.7 ± 2.1%, p < 0.0001; Fig. 6Be). The percentage of elongated NMJs was also significantly increased in the PNC group (42.0 ± 2.6%) compared to sham injured animals (17.3 ± 2.1%, p < 0.0001; Fig. 6Bf). Likewise, the percentage of innervated NMJs was significantly decreased 3 weeks after PNC (58.0 ± 7.0%) compared to sham rats (86.9 ± 2.4%, p = 0.0010; Fig. 6Bg). Moreover, there were significantly fewer multiply innervated NMJs 3 weeks after PNC (14.6 ± 3%) compared to the sham group (46.6 ± 3.8%, p < 0.0001; Fig. 6Bh).

Six weeks after PNC or PNT, the percentage of compact NMJs (74.3 ± 4.0% and 63.6 ± 3.5%, respectively) remained significantly decreased compared to sham rats (87.5 ± 1.1%, p < 0.012; Fig. 6Bi). The percentage of elongated NMJs was also significantly increased 6 weeks after PNC (25.9 ± 3.9%) and PNT animals (35.8 ± 3.4%) compared to sham animals (12.6 ± 1.2%, p < 0.002; Fig. 6Bj). The percentage of innervated NMJs was also significantly decreased 6 weeks after either PNC (79.3 ± 4.8%) or PNT (67.1 ± 9.7%, p = 0.0163) compared to sham rats (92.0 ± 1.9%, P = 0.0163; Fig. 6Bk). Likewise, the percentage of multiply innervated NMJs was decreased 6 weeks after either PNC (35.4 ± 6.3%) or PNT (25.5 ± 7.8%, p = 0.0165) compared to sham rats (53.2 ± 5.4%; Fig. 6Bl).

## Discussion

Extirpative pelvic surgery, particularly RP, is the primary factor contributing to SUI in adult men^24^. This phenomenon arises following the majority of RP cases, and the level of continence regained has been identified as the foremost determinant of post-surgery quality of life following RP^25^. Advances in surgical methods, including robotic-assisted laparoscopic techniques, preservation of cavernosal nerves, reconstruction of the posterior rhabdosphincter, and approaches that spare the Retzius space, have all been introduced with the intention of lowering rates of PPSUI^26^. However, anatomical damage leading to PPSUI can still occur.

During RP, the PN often encounters damage—either a crushing injury during suturing of the dorsal venous complex or a transection injury during apical dissection of the prostate^10^. RP removes or weakens multiple contributors to male continence, including the bladder neck, the urethral suspensory ligaments, and the prostate itself. Consequently, the principal factor influencing continence post-RP becomes the EUS, which is innervated by the PN^9^. In the postoperative phase, the EUS assumes the bulk of urethral resistance, whereas it previously played a role within a more redundant system of continence. The natural recovery of both the EUS and the PN likely accounts for the restoration of continence over the first year after RP for many men^27^.

Several investigations have delved into the advantages of pelvic floor stimulation and direct PN stimulation to facilitate continence recovery after RP^28,29^. Although these studies have revealed perceived improvements in continence, they fall somewhat short in objective outcomes^30^. Consequently, our main goal was to create a model in male rats to facilitate understanding of how PN injury affects SUI. This model can serve as a foundational step for future research investigating the potential of using electrical stimulation or other methods to facilitate neuroregeneration of the PN, as we have previously studied in female rats^22,31^.

In our acute study, we successfully established that exposing the urethral sphincter and subsequently exposing the PN did not lead to any changes in LPP or functional parameters of the EUS. However, we were able to clearly observe immediate and reproducible changes in all parameters upon PNT. Although baseline EUS EMG firing rate of the PNT group was significantly higher compared to the other groups, this discrepancy likely arose due to the diminishing effects of urethane, as the PNT group was assessed later in the sequence after urethane dosing and surgical exposure. Nonetheless, the impact of PNT on firing rate was reinforced by the significantly decreased peak firing rate in the PNT group compared to the NE group, likely due to transection of the PN, as observed in female rats^22,32^. Decreased peak firing rate after PNT indicated that the increased baseline firing rate was not the sole contributor to a decreased firing rate response to LPP testing in the PNT group.

While PNT is a phenomenon that can occur during RP, our chronic study employed PNT primarily as a positive control as we expected little neuroregeneration during the study period based on previous studies in female rats^32,33^. Given this expectation, we limited PNT to animals in the 6-week group to compare recovery of PNC injury, which is more likely to undergo spontaneous recovery in this time period^33^. Our study revealed that animals injured by PNT exhibited minimal urethral neuromuscular function improvement in terms of LPP and EUS EMG firing rate and amplitude compared to the sham control group. This observation is consistent with findings from studies involving PNT injuries in female rats^22,32,33^. On the other hand, we also noted that animals injured by PNC in our study displayed some limited recovery of LPP and EUS EMG parameters 6 weeks after injury with EMG firing rate and amplitude significantly higher than after PNT. This contrasts with the work of Jiang et al., which demonstrated that female rats injured by PNC showed comparable LPP to uninjured control animals as early as 3 weeks post-injury, although EMG firing rate and amplitude remained significantly decreased in injured animals compared to sham controls^33^. We postulate that in male rats, PN efferent nerve fibers must traverse a longer path from the spinal cord to the EUS compared to female rats, which could potentially explain the delayed recovery of LPP and EUS EMG parameters observed in our study compared to similar studies in female rats.

Histological assessment demonstrated increased collagen infiltration, decreased muscle area fraction, and increased collagen to muscle ratio after PNC and PNT at all time points, indicating muscle atrophy and increased scarring in the EUS after injury. Six weeks after injury, PNC resulted in significantly less collagen infiltration and collagen to muscle ratio with decreased muscle area fraction compared to PNT, suggesting partial reinnervation with less muscle atrophy after a crush injury. These histological outcomes are therefore consistent with the neuromuscular function outcomes of LPP and EUS EMG which likewise demonstrated some limited recovery 6 weeks after PNC compared to PNT. This outcome indicates that the PN doesn’t regenerate sufficiently on its own, suggesting that a neuromuscular regenerative therapy has potential to regenerate the PN after injury to prevent muscle atrophy and scarring, as has been demonstrated in female rats^19,28^.

Our histological assessment was based on a custom-developed ImageJ macro for efficient batch-processing, which was rigorously validated against manual quantification using Image-Pro Plus. The validation process, conducted by two researchers across multiple fields and time points, supported the accuracy of our automated analysis. This validated approach was subsequently utilized with confidence, ensuring the reliability and consistency of our findings. Our findings align with observations from prior studies in female rats, reinforcing the consistency and validity of our results^32,34^.

Immunofluorescence staining revealed that the effect of PNC on the EUS was noticeable in reduced compactness and innervation of NMJs. This reduction was evident 4 days and 3 weeks after injury. In contrast, no significant differences in innervation were detected between sham and PNC groups 6 weeks after injury. This observation suggests a recovery process initiated by the PN, leading to reinnervation of the EUS over time. This outcome parallels the functional data we obtained at the same 6-week point, indicating a correspondence between the recovery observed in both the histological and functional aspects. These outcomes find support in the work of Peng et al., whose study highlighted that functional activity, particularly EMG activity, might be influenced by alternative motor nerves that innervate not only the EUS but also adjacent periurethral striated muscles^32^. An example of such motor nerves is the muscular branch of the pelvic nerve responsible for innervating the iliococcygeus/pubococcygeus muscle^32^.

Mersdorf et al. introduced a surgical dissection of the male rat pelvis that allowed for placement of EMG electrodes on the proximal urethra and associated EUS. This study identified urethane as an anesthetic agent that could be used for urethral functional testing without abolishing micturition cycles, and further offered various nerves, including the PN, as worthy of investigating for their role in contribution to male micturition^35^. In both rats and humans, the PN innervates the EUS, making rats an excellent model for investigation, despite the differences between the two species. However, we have only identified two studies since then that studied urethral function of male rats. Khodari et al. evaluated only LPP in animals subjected to cauterization injury of the EUS^11^, while Lee et al. evaluated only LPP in animals subjected to cavernous nerve injury^36^. Both studies sought to evaluate contributors to PPSUI; however, neither included EUS EMG measurements. As such, our study represents a unique exploration of an important contributor to PPSUI and offers a model to explore the benefit of PN stimulation and other therapies for PPSUI patients.

In our study, a single researcher conducted all surgical procedures and performed data analysis for functional testing. This approach ensured a high level of technique consistency. However, it also meant that there was no blinding during the data analysis process, particularly when identifying acceptable LPP peaks and their corresponding EUS EMG frequency and amplitude peaks. The potential for bias from this situation was counteracted by standardization of the technique for application of external bladder pressure as previously described^37,38^. Similarly, when it came to data analysis for histological sections, the same researcher for histology was involved, but measures were taken to minimize possible bias. While the absence of blinding in data analysis could introduce bias, our study proactively countered this by implementing standardized techniques for both functional testing and histological analysis. These measures ensured a rigorous and objective assessment, enhancing the validity of our results.

In contrast to results in female rats^22^, we did not demonstrate continence recovery 6 weeks after injury. We expect this longer regeneration time in males is due to the longer distance of pudendal nerve that needs to regenerate in males. To address this, future research should extend the observation period beyond 6 weeks to explore potential delayed recovery more comprehensively.

The male injury model introduced in this study could serve as a valuable platform for evaluating diverse treatments for PPSUI. Prior studies successfully employed electrical stimulation of the PN to enhance urethral function in a female rat injury model, and this method could be adapted to our male injury model^22^. Additionally, various stem cell treatments, effective in restoring nerve and urethral function in female rats, could be evaluated in the male rat model. Systemically administered mesenchymal stem cells (MSCs) and concentrated conditioned media (CCM) cultured from rat bone marrow derived MSCs demonstrated efficacy in preventing SUI following a multifactorial simulated childbirth injury in a female rat model^31^. This prevention was attributed to the actions of trophic factor secretions, which act by preserving and regenerating the pudendal nerve, promoting elastogenesis, and facilitating enhanced neuromuscular recovery^31^. These and other regenerative approaches merit testing in the male PN injury model.

In conclusion, the present study demonstrates that exposing the urethral sphincter and PN did not yield any alterations in LPP or functional parameters of the EUS in the acute study. Four days and six weeks after injury, PNC induced significant reductions in LPP, EMG amplitude, and firing rate in male rats. Three weeks after injury, both LPP and EMG firing rates decreased in the PNC group. Additionally, 6 weeks after injury demonstrated reductions in LPP, EMG amplitude, and EMG firing rate after both PNC and PNT compared to the sham group. Our histological analysis illuminated the correlation between innervation, injuries, and collagen infiltration, providing valuable insights into the pathophysiology underlying these effects.

## Methods

This study was reviewed and approved (# 00002743) by the Cleveland Clinic Institutional Animal Care and Use Committee. This study was carried out in compliance with the ARRIVE guidelines (Animal Research: Reporting of In Vivo Experiments). All methods, including study design and experimental procedures were conducted in accordance with the relevant guidelines and regulations to ensure transparency, reproducibility, and ethical conduct in the research process.

### Acute study

Six male Sprague–Dawley rats (260–300 g) were subjected to a terminal study of repeated urethral neuromuscular functional recordings after different stages of dissection, such that each animal underwent LPP and EUS EMG testing with the urethra intact, the urethra exposed (UE), the PN exposed bilaterally (NE), and the PN transected bilaterally (PNT). Animals were anesthetized with 2% inhaled isoflurane and were additionally anesthetized with urethane (1.2 g/kg) intraperitoneally. With each rat in a supine position, a midline abdominal incision was made from the upper abdomen to 1 cm below the pubic symphysis, taking care to avoid injury to the penile bulb at the distal end of the incision. The incision was carried through the rectus abdominis, and the bladder and prostate were visualized. A three-point purse-string suture was placed at the dome of the bladder with 4–0 silk suture, and an approximately 1-mm cystotomy was created in the center of the purse-string suture. A flared tip polyethylene (PE-50) tube was inserted into the bladder via the cystotomy to create a suprapubic catheter, and the purse-string was tied to secure the catheter in place. As we have done previously^39^, the catheter was connected to both a pressure transducer (model P122; AstroNova, West Warwick, RI) and a syringe pump (model 200; KD Scientific, New Hope, PA). At this point, the inhaled isoflurane was turned off so that the intraperitoneal urethane represented the sole anesthetic. Bladder pressure was referenced to air pressure at the level of the bladder and was recorded during filling through the suprapubic catheter (5 ml/h).

For LPP testing, we gradually pressed a cotton swab on the bladder when it was approximately half-full until leakage was visible at the meatus, as we have done previously in female rats^39^. At the moment of urine leakage, the cotton swab and all external pressure were removed. If an active bladder pressure contraction was induced by LPP testing, the results were not analyzed, and the test was repeated. The test was repeated 6–8 times in each animal. LPP was calculated as baseline pressure subtracted from peak pressure in the absence of bladder contraction, as we have done previously^39^. The data collected at this point in each rat represented the Intact state.

We then exposed the proximal urethra and the EUS by bluntly dissecting between the apex of the prostate and the pubic bone. Bipolar parallel platinum electrodes (1 mm apart; parallel bipolar electrode, 30211, lot # 243182, L504-01B1, FHC Inc, Bowdoin, ME) for recording EUS EMG were placed externally on the urethra at the location of the EUS. The electrodes were connected to an electrophysiological recording system (PowerLab 8/35, AD Instruments, Colorado Springs, CO; 10 kHz sampling rate with bandpass filtering at 3 Hz–3 kHz). LPP testing was repeated as aforementioned, this time while simultaneously recording EUS EMG and bladder pressure measurements. The data collected at this point in each rat represented the UE state. Each animal was then repositioned in a prone position, and the PNs were accessed bilaterally from a postero-lateral gluteal approach, while the operative area, including the urethra was covered with a moist gauze. The ilium and sacrum were separated slightly until the PNs were visualized. At this point, the animal was repositioned supine, and LPP with simultaneous EUS EMG recordings were again collected. Data collected from this point in each rat represents the NE state.

Each rat was then repositioned in a prone position, and the previously exposed PNs were transected with microdissection scissors, injuring both motor and sensory branches. Each animal was then repositioned supine, and LPP with simultaneous EUS EMG recordings were collected a final time, representing the PNT state. Immediately after the recordings, each rat was euthanized with an overdose (5%) of inhaled isoflurane followed by a bilateral thoracotomy.

### Chronic study

Sixty-seven male Sprague–Dawley rats (250–310 g) were subjected to either PNC, PNT, or sham PN injury. Animals in the PNC and sham groups underwent a terminal study of LPP and EUS EMG recordings 4 days (4d), 3 weeks (3w), or 6 weeks (6w) after injury. Animals in the PNT group only underwent terminal testing 6w after injury to serve as a positive control for PNC 6w after injury, since we expected little to no recovery from the injury in this group. The inclusion of both positive (PNT) and negative (sham) control groups enabled us to determine extent of recovery in the PNC group.

### Pudendal nerve injury

For animals subjected to PNC and PNT, the PN was accessed distal to the spinal column, proximal to the split between motor and sensory branches, using a posterolateral gluteal approach in animals anesthetized with 2% inhaled isoflurane. The ilium and sacrum were separated slightly by a retractor, and the PN was isolated bilaterally. For PNC, the nerves were crushed bilaterally with a Castroviejo needle driver twice for 30 s, as previously described^37,40^. For PNT, the nerves were transected with a 5-mm length of nerve removed to prevent recovery of nerve continuity during healing. After injury, animals in the PNC and PNT groups had their incisions closed with sutures in two layers. Animals subjected to sham injury underwent a midline back incision similar to their PNC and PNT counterparts, but the nerves were not exposed, and the skin was closed with sutures in one layer.

### Leak point pressure (LPP) with simultaneous EUS EMG recordings

After the requisite time following injury (4 days, 3 weeks, or 6 weeks), each animal underwent terminal urethral functional testing as described above for the acute study. In brief, each animal was anesthetized with isoflurane and urethane as described above for the acute study. A suprapubic catheter was inserted, and the urethra was exposed at the EUS as above, after which the isoflurane was turned off so only urethane anesthesia remained. Recording electrodes were placed externally on the urethra at the location of the EUS, and LPP with simultaneous EUS EMG recording was done as described above.

### Histological analysis

Immediately following LPP and EUS EMG functional testing, the animals were euthanized with an isoflurane overdose as described above for the acute study, and the urethra was carefully dissected. It was then flash-frozen in optimal cutting temperature compound and stored at − 80 °C to preserve tissue integrity. Subsequently, the urethral tissues were sectioned (7 µm) and stained with Masson’s trichrome^41^ to assess tissue structure and collagen content. Additionally, 14 µm thick sections were prepared for immunofluorescence staining to investigate innervation of NMJs. Sectioned tissues on slides were fixed in 10% formalin for 15–20 min before staining. Quantitative analysis was performed by independent observers to ensure unbiased evaluation.

### Neuromuscular junction immunofluorescence stain

Immunofluorescence was used to assess the innervation of NMJ in the EUS qualitatively and quantitatively as modified from previous research^39^. Primary antibodies, mouse monoclonal anti-neurofilament 68 clone NR4, and mouse monoclonal anti-neurofilament 200 clone N52 (Sigma-Aldrich, St. Louis, MI, USA) were combined in a 1:400 dilution. The secondary antibody, Alexa Fluor donkey anti-mouse 488 IgG (A21202, Invitrogen ThermoFisher) was used in a 1:1000 dilution to label nerve fibers. Additionally, Tetramethylrhodamine alpha-Bungarotoxin (T1175, Invitrogen ThermoFisher) in 1:250 dilution identified acetylcholine receptors (AChR) of the NMJ, and Alexa Fluor 350 phalloidin (A22281, Invitrogen ThermoFisher) in 1:40 dilution identified striated muscle of the EUS. Tissue sections were scanned with a digital Leica DM6 slide scanner system using a 40 × objective, and representative images were captured for each section.

### Data analysis

During LPP testing, bladder pressure, EUS EMG amplitude, and EUS EMG firing rate were assessed as we have done previously in female rats^39^. In brief, 3 representative one-second intervals were captured for peak and baseline values during LPP testing for each animal in each group at each timepoint. Values of LPP, EUS EMG amplitude, and EUS EMG firing rate at baseline prior to LPP testing, at peak pressure of LPP testing, and the difference between them, which was taken as the response to LPP testing, were calculated as done previously^39^. In this case, EMG firing rate is an average of the composite of multiple muscle fibers. LPP was defined as the difference between peak and baseline pressure during LPP testing.

Masson’s trichrome stain was used for both qualitative and quantitative histological analysis. For analysis, ImageJ 1.54f version Java 1.8.0_322 (NIH, USA) software with a developed macro was used for batch-processing. The Color Deconvolution tool in ImageJ separated the image into its three-color components^42^. To isolate collagen fibers, the blue component was manually threshold-adjusted to accurately cover the blue area, and the corresponding area was measured for collagen quantification. Similarly, the red component was analyzed to determine the muscle tissue area. Pixel areas were then used to calculate the collagen infiltration percentage (collagen fiber area/total tissue area), muscle tissue percentage (muscle tissue area/total tissue area), and the collagen-to-muscle ratio (collagen fiber area/muscle tissue area). An ImageJ macro was written for batch-processing to expedite the process, which automatically looped through all images in a folder and executed the necessary commands, pausing for user input during the threshold adjustment step. Manual quantification with Image-Pro Plus (Version 7.0, Media Cybernetics Inc., Rockville, MD) as previously described^43^ was used to validate the automated ImageJ analysis.

The assessment of EUS NMJ was based on the following criteria: (a) Compactness: Whether the NMJ had well-defined, compact edges (Compact or elongated). (b) Multiple innervating axons: Whether there was more than one axon innervating a NMJ (yes or no). (c) Innervation: Whether the NMJ was innervated by a single axon or multiple axons (single or poly innervation). For each group (sham, PNC, and PNT), 7–10 animals were used, and the percentages of compact NMJ (stained in red) relative to the total NMJ were determined for each specimen. The same assessment was done for innervated and multiply innervated NMJ. The percentage values were calculated for 12 different fields per specimen, ensuring a comprehensive evaluation of EUS NMJ innervation.

### Statistical analysis

For both the functional and histological analysis, mean values were calculated for each animal at each testing interval, and these means were used to calculate the mean and standard error of the mean for each group. In the acute study, the Kruskal–Wallis test followed by Dunn’s multiple comparisons test was used to compare LPP, EUS EMG amplitude, and EMG firing rate between four groups (Intact, UE, NE, and PNT). In the chronic study and histology, an unpaired two-tailed *t*-test was used to compare outcomes between the sham and PNC groups 4 days and 3 weeks after injury. Furthermore, ordinary one-way analysis of variance (ANOVA) followed by the Tukey’s multiple comparisons test was used to compare outcomes among the sham, PNC, and PNT groups 6 weeks after injury. A significance level of P < 0.05 was used to indicate a statistically significant difference between groups in all analyses.

### Ethics statement

This research received approval from the Institutional Animal Care and Use Committee of the Cleveland VA Medical Center.

## Data Availability

The dataset generated and analyzed during this study is available upon reasonable request from the corresponding author.
